# Predicting the optimal concentration of remifentanil for skull pin fixation with hemodynamic and analgesia nociception index monitoring

**DOI:** 10.1038/s41598-024-56283-z

**Published:** 2024-03-18

**Authors:** Yi-Wei Kuo, Ying-Tzu Chen, Ann-Shung Lieu, Meei-Shyuan Lee, Yu-Feng Su, Hou-Chuan Lai, Zhi-Fu Wu

**Affiliations:** 1 Department of Anesthesiology, Kaohsiung Medical University Hospital, Kaohsiung Medical University, No. 100, Tzyou 1st Rd., Sanmin Dist., Kaohsiung City, 80756 Taiwan, ROC; 2 Department of Surgery, Division of Neurosurgery, Kaohsiung Medical University Hospital, Kaohsiung Medical University, Kaohsiung, Taiwan, ROC; 3 School of Public Health, National Defense Medical Center, Taipei, Taiwan, ROC; 4 Department of Anesthesiology, Tri-Service General Hospital and National Defense Medical Center, #325, Section 2, Chenggung Road, Neihu 114, Taipei, Taiwan, ROC; 5 Department of Anesthesiology, Faculty of Medicine, College of Medicine, Kaohsiung Medical University, Kaohsiung, Taiwan, ROC; 6 Center for Regional Anesthesia and Pain Medicine, Wan Fang Hospital, Taipei Medical University, Taipei, Taiwan, ROC

**Keywords:** Remifentanil, Analgesia nociception index, Skull pin fixation, Intracranial surgery, Medical research, Neurology

## Abstract

Inadequate antinociception during skull pin fixation may cause hemodynamic instability in intracranial surgery. The optimal concentration of remifentanil to provide adequate antinociception and stable hemodynamics during skull pin fixation under analgesia nociception index monitoring is unknown. This study is to assess the 90% effective concentration of remifentanil for skull pin fixation under hemodynamic and analgesia nociception index monitoring. Twenty-six patients were enrolled for intracranial surgery, anesthesia was induced and maintained under total intravenous anesthesia using target-controlled infusion for remifentanil and propofol under analgesia nociception index and bispectral index monitoring. Skull pin fixation was performed at different effect-site concentrations of remifentanil required for Dixon's up-and-down method with a step size of 0.5 ng/ml under bispectral index 40–60. Inadequate antinociception is defined when either ANI < 30 or > 20% in hemodynamic changes from baseline (e.g. heart rate > 100 beats/min, or blood pressure > 180/100 mmHg) and the effect-site concentration of remifentanil is considered as failure. It is considered success as ANI > 30 and < 20% hemodynamic changes from baseline simultaneously. Seven pairs of failure/success were used for probit analysis. The 90% effective concentration of remifentanil for skull pin fixation with adequate antinociception and hemodynamic stability was 4.7 ng/ml.

## Introduction

Skull pin fixation for craniotomy elicits a significant hemodynamic response despite the optimal depth of general anesthesia (GA)^1^. The changes in heart rate (HR) and blood pressure (BP) reflect an autonomic (sympathetic) response to noxious stimulation from skull pin fixation, as the scalp and the periosteum are richly innervated with nerve fibers^1^. Anesthesiologists may encounter hemodynamic changes that require pharmacological intervention due to a noxious stimulus resulting from skull pin fixation. Acute arterial hypertension may lead to intracranial hemorrhage and induce intracranial hypertension and cerebral edema in patients with intracranial tumor^2,3^. Thus, various strategies such as local anaesthetics, opioids, and scalp blocks have been used to blunt hemodynamic responses induced by skull pin fixation^2,4–7^. Though scalp block or local infiltration may provide adequate analgesia and hemodynamic stability, it requires extra time and is not our routine practice^2^. Total intravenous anesthesia (TIVA) is a standard and widely used method of GA, especially for evoked potential monitoring during intracranial surgery^2^. Due to its pharmacodynamic and pharmacokinetic characteristics, such as a very short context-sensitive half-time and minimal effects on cardiovascular system, remifentanil is a commonly used opioid in conjunction with propofol for TIVA^2,8^.

The severity of pain and its clinical symptoms are difficult to assess in the absence of objective intraoperative analgesia monitor. Some nociception/anti-nociception balance techniques of monitoring [such as surgical pleth index (SPI) or analgesia nociception index (ANI)] were successfully introduced, which proved the utility in neuroanaesthesia regarding efficacy of pain perception^9–13^. The ANI is a more common index that measures high-frequency component of heart rate variability on a scale from 0 (maximum of nociception) to 100 (complete analgesia)^9–12^. Based on the published studies^9–12^, an ANI of between 50 and 70 may correspond to adequate antinociception. Two ANI values provided by the monitor, mean-ANI (ANI_m_), an average calculated over the previous 4 min, and instant-ANI (ANI_i_), an average calculated over a shorter period of time (64 s)^14^. For skull pin fixation, Kommula et al. found that ANI values were well correlated with hemodynamics^15^. The ANI is superior in detecting painful stimulations compared to HR and mean arterial pressure (MAP) during propofol and remifentanil anesthesia^10^. Sabourdin et al. demonstrated that ANI guidance resulted in lower remifentanil consumption compared with standard practice under propofol and remifentanil anesthesia^11^.

There are several options to prevent hemodynamic perturbation during skull pinning as above mentioned, however, the optimal effective concentration (EC) of remifentanil for skull pin fixation without inadequate antinociception and hemodynamic instability has not been thoroughly investigated. This study was designed to estimate the EC_90_ of remifentanil for blunting cardiovascular responses to skull pin fixation during intracranial surgery under remifentanil/propofol TIVA with ANI and bispectral index (BIS) monitoring.

## Materials and methods

This study was conducted at the Kaohsiung Medical University Hospital (KMUH), Kaohsiung, Taiwan, Republic of China. The Institutional Review Board of the KMUH approved this study (KMUHIRB-F(I)-20210156), and all methods were performed in accordance with the relevant guidelines and regulations. Written informed consent was obtained from all patients. This study was first registered at the ClinicalTrials.gov (www.clinicaltrials.gov) on 18/11/2021, with registration number NCT05125328. Twenty-six adult patients scheduled for elective intracranial surgery with skull pin fixation under intubated TIVA using a target-controlled infusion (TCI) systems (TCI, Fresenius Orchestra Primea; Fresenius Kabi AG, Bad Homburg, Germany) were enrolled in this study. The eligible patients were aged 20 to 80 years with ASA physical status I–III. The exclusion criteria for this study were as following: patients with ASA physical status ≥ IV, patients with major comorbid diseases under beta-adrenergic blocker use, patients with a pacemaker or a significant arrhythmia (eg, atrial fibrillation), patients with chronic pain, emergent surgery, and allergy to propofol or remifentanil.

All patients were fasted overnight before the procedure, and no medications were administrated before the induction of anesthesia. Each patient received standard monitoring, including electrocardiography (lead II), noninvasive BP testing, pulse oximetry, end-tidal carbon dioxide (EtCO_2_) measurement, and direct radial ABP monitoring. In addition, all patients underwent monitoring for the BIS (BIS™ Complete 2-Channel Monitor, COVIDIEN, Boulder, CO, USA) and ANI (Physiodoloris®, MDoloris Medical Systems, Loos, France). Participants were preoxygenated with 6 l/min 100% oxygen via a facial mask to achieve peripheral oxygen saturation of 99- 100% before induction.

Patients were induced with an effect-site concentration (Ce) of 2.0–4.0 ng/ml of remifentanil (50 mcg/ml, Minto model) and Ce 3.0–6.0 mcg/ml of propofol (10 mg/ml; Schnider model) with continuous infusion using two separate TCI pumps. Rocuronium (0.6 mg/kg) was administered after loss of consciousness in all patients to facilitate endotracheal intubation. Anesthesia was maintained with an oxygen flow of 0.3 l/min and mixed air 0.7 l/min. The EtCO_2_ was maintained at 35–45 mmHg by adjusting the ventilation rate and maximum airway pressure < 30 cmH_2_O. The intraoperative administration of propofol and remifentanil was guided by maintaining the BIS value at 40–60 and a mean (4-min moving average) ANI (ANI_m_) of 50–70 during surgery.

The target Ce of remifentanil for skull pin fixation was adjusted by using Dixon’s up–and down sequential method^16,17^. Skull pin fixation was performed when the preset remifentanil concentration was reached and persisted at least 2 min, and the Ce of remifentanil for the first subject was set to 6.0 ng/ml based on our clinical experience. If inadequate antinociception (ANI < 30)^18^ or hemodynamic instability [> 20% increase in hemodynamic changes from baseline such as HR and mean arterial pressure (MAP)] or HR > 100 beats/min (bpm) or ABP > 180/100 mmHg during skull pin fixation was defined as failure; otherwise, the setting was considered successful without abovementioned situations. The next setting of remifentanil concentration was predetermined by the response of previous patient with a higher or lower dose (0.5 ng/ml as a step size). After a failure trial, the target concentration of remifentanil was increased by 0.5 ng/ml for next patient. Conversely, if no inadequate antinociception or hemodynamic instability was observed, the remifentanil concentration was decreased by 0.5 mg/ml for next patient. We recorded the Ce of remifentanil, Ce of propofol, HR, MAP, BIS, and ANI values at 5 time points as following: T0: baseline (at initial induction of GA before loss of consciousness); T1: 2 min before skull pin fixation; T2: during skull pin fixation; T3: 5 min after skull pin fixation; T4: 15 min after skull pin fixation. For patients with HR > 100 bpm or ABP > 180/100 mmHg during anesthesia, beta-blockers or anti-hypertensive agents were administered. For patients with HR < 50 bpm or ABP < 90/50 mmHg during anesthesia, atropine or ephedrine was given. After surgery, all patients were transferred to the intensive care unit. All patients were anesthetized by one anesthesiologist, and another investigator assessed them for the presence of a successful or failure response to skull pin fixation. Neither the surgeon performing skull pin fixation nor the patient was aware of the remifentanil/propofol Ce during skull pin fixation.

Demographic data were collected and are presented as mean and standard deviation (SD). Dixon’s up-and-down method needs at least six pairs of failure/success for statistical analysis, and sample size came from the basis of Dixon’s method^14^. Seven pairs of failure/success were used for probit analysis for this study, which enabled us to derive the target remifentanil concentration for skull pin fixation with 95% confidence limits of the mean. The EC_50_ and EC_90_ were estimated using the probit model. All the variables during the study period, including ANI, BIS, HR, MAP, Ce of propofol and remifentanil at different time points were analyzed by a repeated measures analysis of variance, and the Turkey procedure was conducted as appropriate, correcting for multiple comparisons. Linear regression was performed to determine the relationship among ANI, HR, MAP, BIS or Ce of propofol and remifentanil. A P value of < 0.05 was considered significant. The statistical tests were performed using a SPSS Statistics Version 28.0 (IBM Corp., Armonk, NY, USA).

### Ethics statement

This study was conducted at the Kaohsiung Medical University Hospital (KMUH), Kaohsiung, Taiwan, Republic of China. The Institutional Review Board of the KMUH approved this study (KMUHIRB-F(I)-20210156), and all methods were performed in accordance with the relevant guidelines and regulations. Written informed consent was obtained from all patients. This study was first registered at the ClinicalTrials.gov (www.clinicaltrials.gov) on 18/11/2021, with registration number NCT05125328.

## Results

**Figure 1:**
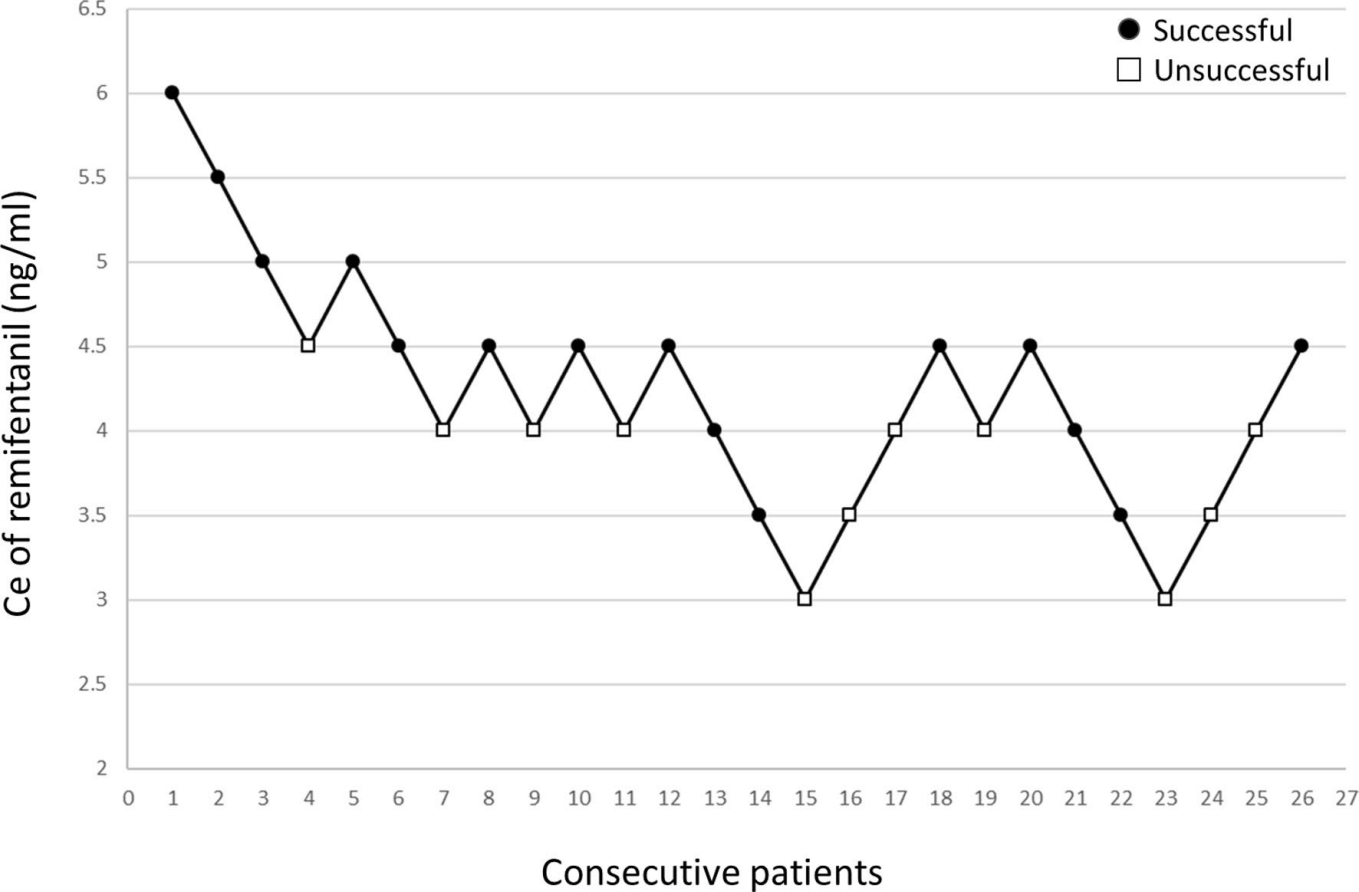
The Ce of remifentanil in the 26 consecutive patients in whom the skull pin fixation was attempted. Each patient's data are represented with a circle or a square; a filled circle (●) means successful skull pin fixation, a hollow square (□) means unsuccessful skull pin fixation.

Twenty-six patients were included in this study. The plots of Ce of remifentanil associated with success or failure of skull pin fixation for each consecutive patient were shown in the Fig. 1. The patients’ demographic data and the perioperative events were presented in Table 1. There were 7 male and 19 female patients with age of 59.5 ± 10.4 years, height of 159.3 ± 7.3 cm, weight of 64.9 ± 15.9 kg, anesthesia time of 299.2 ± 113.5 min, and operation time of 218.7 ± 106.2 min. There was no patient received anti-hypertensive agents or beta-blockers after adjusting remifentanil dosage during this trial. Eight patients received ephedrine due to post-induction hypotension before this trial (Table 1).

**Table 1:** Baseline characteristics and perioperative events of patients.

Age (year)	59.5 (10.4)
Sex (Male/Female)	7/19
Height (cm)	159.3 (7.3)
Weight (kg)	64.9 (15.9)
Tumor location	
Pituitary	9
Frontal	6
Cerebellar	3
Parasagittal	2
Parietal	2
Skull base	2
Occipital	1
Temporal	1
Anesthetic time (mins)	299.2 (113.5)
Operation time (mins)	218.7 (106.2)
Ephedrine	8

Values are expressed as mean (standard deviations; SD) or number except for sex.

Eight patients received ephedrine due to post-induction hypotension.

Table 2 reported the Ce of propofol and remifentanil, BIS values, HR, and MAP during study. Ce of propofol/remifentanil at T0 (3.7 ± 1.1 mcg/ml; 3.1 ± 0.9 ng/ml) was significantly higher than that at T3 (2.2 ± 0.6 mcg/ml; 1.9 ± 0.5 ng/ml) and T4 (2.3 ± 1.2 mcg/ml; 1.1 ± 0.8 ng/ml). Ce of propofol at T0 (3.7 ± 1.1 mcg/ml) was significantly higher than that at T1 (2.3 ± 0.7 mcg/ml) and T2 (2.3 ± 0.7 mcg/ml). Ce of remifentanil at T1/T2 (4.1 ± 0.9; 4.2 ± 0.7 ng/ml) was significantly higher than that at T3 and T4 (P < 0.05). BIS level at T0 (92.7 ± 7.2) was significantly higher than BIS level at T1 (49.6 ± 10.5), T2 (46.0 ± 6.6), T3 (44.6 ± 8.6), and T4 (47.0 ± 8.5). In addition, the results of T0 data (BIS of 92.7 ± 7.2 under propofol Ce of 3.7 ± 1.1 mcg/ml) were at initial induction of GA before loss of consciousness. HR at T0/T1 (75.3 ± 14.4; 72.2 ± 13.9 bpm) was significantly higher than HR at T3 (68.3 ± 14.1 bpm, P < 0.05). MAP at T2 (102.3 ± 16.6 mmHg) was significantly higher than MAP at T1 (92.2 ± 13.0 mmHg) and T3 (84.9 ± 13.3 mmHg) (P < 0.05) (Table 2). Finally, the Ce of propofol and remifentanil, BIS values, HR, and MAP during study revealed significantly different at different time points (P < 0.001).

**Table 2:** Propofol and remifentanil effective concentration (Ce), bispectral index (BIS) values, heart rate (HR), and mean arterial pressure (MAP) during skull pin fixation.

	T0	T1	T2	T3	T4	P value
Ce of propofol (mcg/ml)	3.7 ± 1.1^#^	2.3 ± 0.7^#^	2.3 ± 0.7^#^	2.2 ± 0.6^#^	2.3 ± 1.2^#^	< 0.001
Ce of remifentanil Ce (ng/ml)	3.1 ± 0.9*^!^	4.1 ± 0.9^%+^	4.2 ± 0.7^▼&^	1.9 ± 0.5*^▼+^	1.1 ± 0.8^%&!^	< 0.001
BIS	92.7 ± 7.2^#^	49.6 ± 10.5^#^	46.0 ± 6.6^#^	44.6 ± 8.6^#^	47.0 ± 8.5^#^	< 0.001
HR (bpm)	75.3 ± 14.4*	72.2 ± 13.9^+^	70.2 ± 12.5	68.3 ± 14.1*^+^	68.5 ± 12.6	< 0.001
MAP (mmHg)	101.3 ± 12.4	92.2 ± 13.0^$^	102.3 ± 16.6^$▼^	84.9 ± 13.3^▼^	94.1 ± 15.7	< 0.001

Ce indicates effect-site concentration.

T0: baseline (at initial induction of general anesthesia before loss of consciousness); T1: 2 min before pin fixation; T2: during pin fixation; T3: 5 min after pin fixation; T4: 15 min after pin fixation.

^#^P < 0.05 as T0 versus other time points.

^@^P < 0.05 as T0 versus T2.

*P < 0.05 as T0 versus T3.

^!^P < 0.05 as T0 versus T4.

^$^P < 0.05 as T1 versus T2.

^+^P < 0.05 as T1 versus T3.

^%^P < 0.05 as T1 versus T4.

^▼^P < 0.05 as T2 versus T3.

^&^P < 0.05 as T2 versus T4.

Table 3 compared HR, MAP, ANI, BIS, and Ce of propofol and remifentanil between successful and failure patients during skull pin fixation. Successful patients with higher remifentanil Ce (4.5 ± 0.6 vs. 3.8 ± 0.5, P = 0.004) and ANI_i_ (51.1 ± 16.9 vs. 27.2 ± 7.9, P < 0.001) compared with failure patients. There was no significant difference in HR, MAP, BIS, and propofol Ce between the successful and failure patients (Table 3).

**Table 3:** The comparison of hemodynamics, analgesia nociception index, bispectral index, and effect-site concentrations of propofol and remifentanil between successful and failure patients during skull pin fixation.

	Successful	Unsuccessful	P value
N	15	11	
HR	71.1 ± 11.8	69 ± 13.4	0.683
MAP	97.6 ± 15.5	108.6 ± 15.8	0.101
ANI_i_	51.1 ± 16.9	27.2 ± 7.9	< 0.001*
BIS	44.9 ± 5.8	47.5 ± 7.4	0.359
Ce of propofol	2.4 ± 0.6	2.2 ± 0.7	0.298
Ce of remifentanil	4.5 ± 0.6	3.8 ± 0.5	0.004*

Ce indicates effect-site concentration; ANIi indicates an instantaneous value calculated as a mobile mean of ANI over 64 s of monitoring.

*BIS* bispectral index.

**Figure 2:**
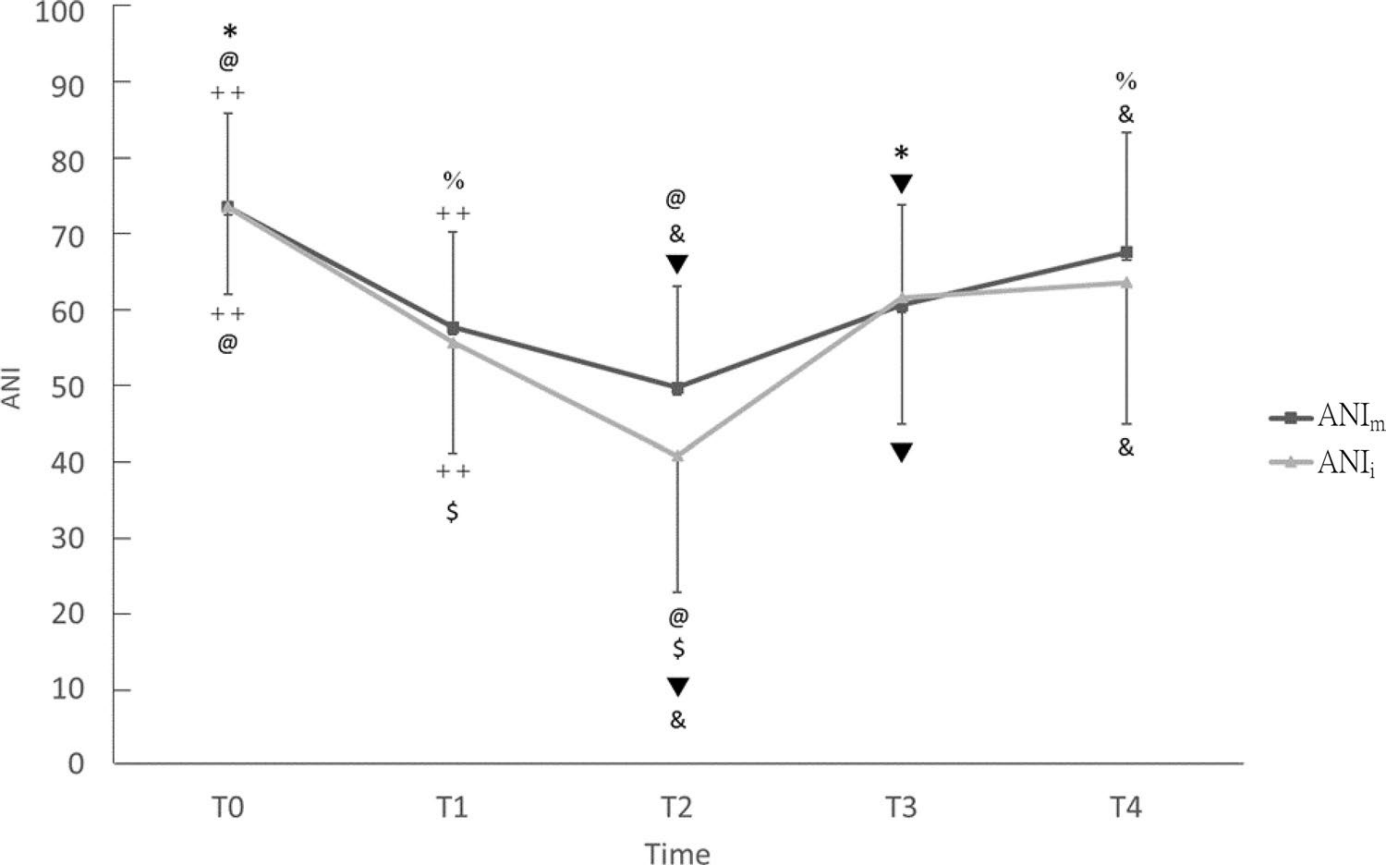
Comparison of analgesia nociception index (ANI) during skull pin fixation at different time points (T0: baseline; T1: 2 min before skull pin fixation; T2: during skull pin fixation; T3: 5 min after skull pin fixation and T4: 15 min after skull pin fixation). ANI_m_ indicates a mean value calculated as a mobile mean of ANI over 4 min of monitoring; ANI_i_ indicates an instantaneous value calculated as a mobile mean of ANI over 64 s of monitoring. ^++^P < 0.05 as T0 versus T1; ^@^P < 0.05 as T0 versus T2; *P < 0.05 as T0 versus T3; ^$^P < 0.05 as T1 versus T2; ^%^P < 0.05 as T1 versus T4; ^▼^P < 0.05 as T2 versus T3; ^&^P < 0.05 as T2 versus T4.

Figure 2 compared ANI values at each time point. ANIi at T1/T3/T4 (56.4 ± 14.7; 62.4 ± 16.8; 64.1 ± 18.9) was significantly higher than that at T2 (41.0 ± 18.2; P < 0.05). ANI_i_ at T0 (73.7 ± 11.6) was significantly higher than that at T1 and T2 (P < 0.05). In addition, ANI_m_ at T0 (74.1 ± 12.5) was significantly higher than that at T1 (57.6 ± 12.7), T2 (50.0 ± 13.5), and T3 (61.3 ± 13.3). ANI_m_ at T4 (68.3 ± 15.9) was significantly higher than that at T1 and T2 (P < 0.05), and T3 was significantly higher than that at T2 (P < 0.05; Fig. 2).

**Figure 3:**
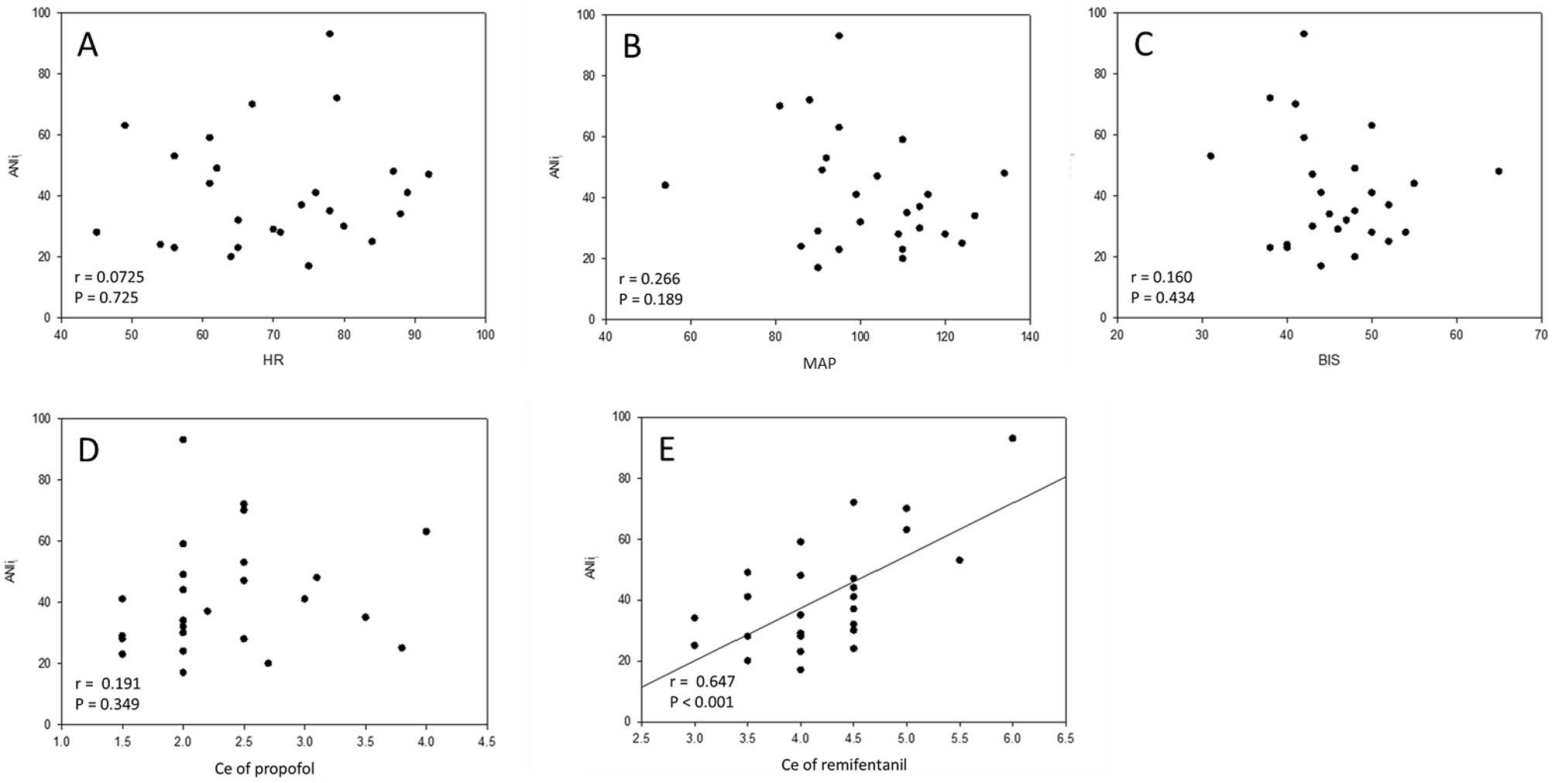
Scatter plots demonstrated the correlation between ANI_i_ and (**A**) heart rate (HR), (**B**) mean arterial pressure (MAP), (**C**) bispectral index (BIS), (**D**) Ce of propofol, and (**E**) Ce of remifentanil. ANI_i_ was significantly correlated with remifentanil Ce (r = 0.647, P < 0.001), but not HR, MAP, BIS, or propofol Ce. ANI_i_ indicates an instantaneous value calculated as a mobile mean of ANI over 64 s of monitoring. Ce indicates effect-site concentration.

Figure 3 scatter plots demonstrated the corelation among HR, MAP, BIS, Ce of propofol and remifentanil, and ANI_i_. ANI_i_ was significantly correlated with remifentanil Ce (r = 0.647, P < 0.001), but not HR, MAP, BIS, or propofol Ce.

**Figure 4:**
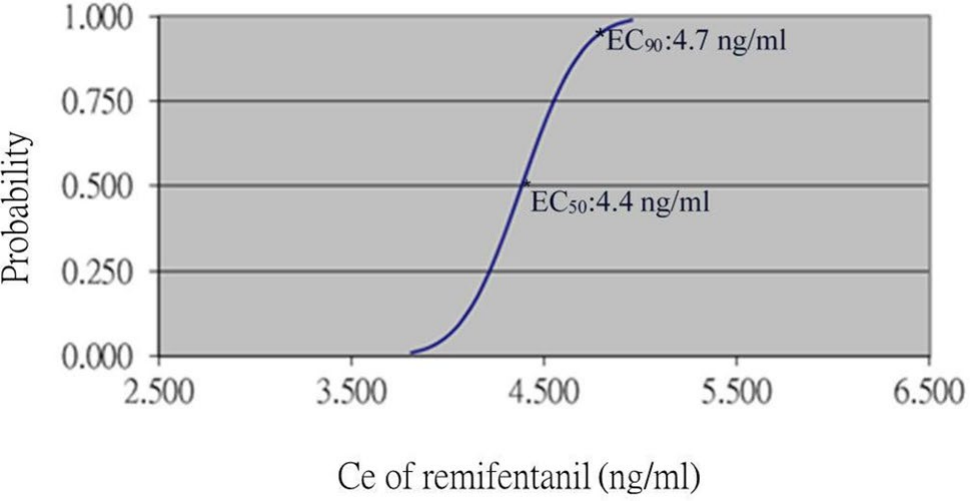
Dose–response curve for remifentanil from the probit analyses of individual concentrations and the respective patient reactions to skull pin fixation. The concentrations of remifentanil at which there were 50% and 90% probabilities of successful skull pin fixation were 4.4 ng/ml and 4.7 ng/ml, respectively. Seven pairs of failure/success were used for probit analysis.

Figure 4 showed the EC_50_ for adequate antinociception and cardiovascular response inhibition to skull pin fixation using remifentanil was 4.4 ng/ml and EC_90_ was 4.7 ng/ml. Seven pairs of failure/success were used for probit analysis.

## Discussion

The major finding in our study is that the effective Ce of remifentanil at which there were 50% and 90% probabilities of successful skull pin fixation were 4.4 ng/ml and 4.7 ng/ml, respectively. Accordingly, we suggest that skull pin fixation can be performed 90% patients without noxious stimulation-induced cardiovascular response after adjusting remifentanil Ce of 4.7 ng/ml under remifentanil/propofol TCI and ANI/BIS monitoring. In addition, ANI monitoring is more sensitive on remifentanil requirement than hemodynamics and BIS during skull pin fixation.

There is no previous literature objectively evaluating the autonomic response to noxious stimulation from skull pin application using remifentanil/propofol TCI under ANI and BIS monitoring. Previous studies have investigated the most effective method of alleviating hemodynamic responses to skull pin fixation^1,19–22^. The authors introduced and compared various strategies, such as intravenous opioids, local anesthetic infiltration at the pin sites, a combination of intravenous fentanyl and local anesthetic infiltration, and scalp nerve blockade. However, no consensus exists to guide anesthesiologists in attenuating cardiovascular responses to skull pin fixation. Intravenous fentanyl injection alone may not be sufficiently effective in many cases, and local anesthetic infiltration may not always be effective because sometimes the exact pin sites may not match the infiltrated scalp area. Moreover, scalp nerve block is not always effective, and its performance requires extra time and training^2^. As mentioned previously, remifentanil has excellent characteristics and is commonly used, along with propofol, by anesthesiologists for TIVA with a TCI system during neurosurgery. If the Ce of remifentanil that effectively reduces hemodynamic responses to skull pin fixation is attained, the anesthesiologists can maintain a stable hemodynamic status with fewer drugs and use a simpler approach.

Previously, two similar studies were conducted to determine the EC_50_ and EC_90_ of remifentanil necessary to minimize the cardiovascular changes due to skull pin fixation under TIVA with remifentanil and propofol under BIS but without ANI monitoring^2,23^. Lee et al. used the biased coin up-and-down design sequential method to calculate the remifentanil EC_50_ of 5.33 ng/ml, the EC_90_ of 6.48 ng/ml and EC_95_ of 6.74 ng/ml^2^. Do et al. used the Dixon up-and-down sequential allocation method to find the remifentanil EC_50_ of 2.90 ng/ml and the EC_95_ of 4.28 ng/ml^23^. Our results were similar with Do et al. reporting that the EC_95_ of remifentanil was 4.28 ng/ml based on the same statistic method, Dixon up-and-down sequential allocation method. On the other hand, Lee et al. used the biased coin up-and-down design sequential method and kept BIS values at between 40 and 50 perioperatively, and their values were higher than Do et al.'s and our results. However, they did not use ANI monitoring during study. Though Lee et al. showed that the Ce of remifentanil were higher than our results (6.5 vs. 4.7 ng/ml) during skull pin fixation, it might be due to gender-related differences in the Ce of remifentanil based on our majority of female patients (43.6% vs. 73.1%), and female patients might be more sensitive to ANI monitoring^24^ and require less opioid dosage^25^. However, further research on gender issue is necessary. In this study, there were total 11 failure patients, two (2/11; 18.2%) failure patients with ANI > 30 but hemodynamic change > 20%. On the other hand, nine (9/11; 81.8%) failure patients with ANI < 30. Among them, three patients (3/11; 27.3%) with both ANI < 30 and cardiovascular changes > 20%, and six patients (6/11; 54.5%) with ANI < 30 and cardiovascular changes < 20%. We also found that the ANI_i_ was significantly correlated with the Ce of remifentanil (r = 0.647, P < 0.001), but not HR, MAP, BIS, or propofol Ce (Fig. 3). Therefore, our results might provide more accurate information to prevent noxious stimulation during skull pin fixation.

Several studies have evaluated ANI in identifying the pain during the intraoperative phase. ANI decreased with airway manipulation, skin incision and increased with fentanyl administration^26^. However, the use of ANI to provide clinical benefits, such as decreased intraoperative opioid use, postoperative opioid use, and postoperative pain compared to standard practices appeared controversial^27^. In the review, ANI-guided intraoperative opioid consumption might vary based on the type and length of the surgery associated with nociceptive stimuli, the pharmacological properties of the different anesthetics (such as propofol versus inhalation, or continuous infusion of opioid such as remifentanil versus bolus of fentanyl), and sample sizes^27^. Jeanne et al. found that ANI monitoring was more sensitive than hemodynamic parameters to moderate noxious stimuli under propofol anesthesia in patients undergoing laparoscopic surgery^28^. In addition, Daccache et al. have shown that ANI can be used to adequately guide intraoperative remifentanil administration during vascular surgery under TIVA with propofol^29^. Theerth et al. successfully used ANI for pain monitoring in skull pin fixation under regional analgesic techniques, but not propofol/remifentanil TIVA^1^. Here, we first used propofol/remifentanil TIVA under ANI and BIS monitoring for pain monitoring in skull pin fixation. And we found that successful patients with significantly higher Ce of remifentanil, ANI_m_, and ANI_i_ compared with failure patients (Table 3). However, there was no significant difference in HR, MAP, BIS, and propofol Ce between the successful and failure patients.

There were some limitations in this study. First, the sample size was small with the majority of female patients, and the results were only applicable for the Chinese and not appropriate for other populations. Second, increased Ce of propofol might reduce EC_50_ and EC_90_ of remifentanil during procedure^30^. Many studies have been conducted to find the Ce of propofol required for appropriate unconsciousness when TIVA was performed. Ithnin et al. reported that Ce of propofol for adequate tracheal intubating condition was 3.0 mcg/ml with Ce of remifentanil 4.41 ng/ml^31^. Do et al. reported that Ce of propofol was maintained at 2.0 mcg/ml to keep BIS levels between 40 and 60 during head holder pinning^23^. In this study, the Ce of propofol during skull pin fixation (T2) was maintained at 2.3 ± 0.7 mcg/ml to keep BIS levels between 40 and 60. Our setting was consistent with Do et al. study. On the other hand, Lee et al. showed that both the Ce of propofol at 2.9 ± 0.7 mcg/ml (to keep BIS levels between 40 and 50), and the Ce of remifentanil were higher than our results during skull pin fixation, it might be due to age/gender-related differences in the Ce of propofol/remifentanil based on our majority of older female patients^24,32^. However, age might not affect the Ce of remifentanil during surgery^33^. Further studies are needed for checking effects of age and gender to Ce of propofol/remifentanil. Third, in our clinical practice, we didn’t routinely perform pin‑site infiltration or scalp block for patients. However, local anesthetic infiltration might not always be effective because sometimes the exact pin sites might not match the infiltrated scalp area^1^. Moreover, scalp nerve block is not always effective^34^, and its performance required extra time and training^2^. Further research is necessary. Fourth, there were 6 failed patients who showed ANI < 30 and cardiovascular changes < 20%. These patients might be regarded as success when we applied them with only hemodynamic criteria. However, we increased a step size according to inadequate antinociception based on our protocol. Fortunately, increasing a step size resulted in success without any side effect following these 6 failed patients. Fifth, our protocol used ANI-guided remifentanil administration based on our clinical experience, but not SPI or pupillometry-guided, which might be complicated for inexperienced anesthesiologists to practice^8^. However, the ANI monitor is a more common device in our hospital. Finally, we failed to aim to keep BIS above 45 during surgery, and BIS < 45 might result in bradycardia and hypotension and affect nociception/anti-nociception balance^35^. Further research is necessary to check the relationship of BIS values and Ce of propofol/remifentanil for nociception/anti-nociception balance.

## Conclusions

Under ANI monitoring, adjustment of the Ce of remifentanil to approximately 4.7 ng/ml at least 2 min before skull pin fixation could blunt noxious stimulation and provide stable cardiovascular responses in 90% of intracranial surgery ASA I–III patients while propofol TCI titrated to maintain BIS between 40 and 60.

## Data Availability

The original contributions presented in the study are included in the article, and further inquiries can be directed to the corresponding author.

## Abbreviations

ABP: Arterial blood pressure
ANI: Analgesia nociception index
ANI_i_: Instant-analgesia nociception index
ANI_m_: Mean-analgesia nociception index
ASA: American Society of Anesthesiology
BIS: Bispectral index
BP: Blood pressure
Ce: Effect-site concentration
EC: Effective concentration
EtCO_2_: End-tidal carbon dioxide
GA: General anesthesia
HR: Heart rate
IRB: Institutional review board
KMUH: Kaohsiung Medical University Hospital
MAP: Mean arterial pressure
SD: Standard deviation
SPI: Surgical pleth index
TCI: Target-controlled infusion
TIVA: Total intravenous anesthesia
